# Optical Method for Estimating the Chlorophyll Contents in Plant Leaves

**DOI:** 10.3390/s18020650

**Published:** 2018-02-22

**Authors:** Madaín Pérez-Patricio, Jorge Luis Camas-Anzueto, Avisaí Sanchez-Alegría, Abiel Aguilar-González, Federico Gutiérrez-Miceli, Elías Escobar-Gómez, Yvon Voisin, Carlos Rios-Rojas, Ruben Grajales-Coutiño

**Affiliations:** 1 Tecnológico Nacional de México, 29000 Tuxtla Gutiérrez, Chiapas, Mexico; mperez@ittg.edu.mx (M.P.-P.); asancheza@gdl.cinvestav.mx (A.S.-A.); fgmiceli@gmail.com (F.G.-M.); enescobarg@hotmail.com (E.E.-G.); crios@ittg.edu.mx (C.R.-R.); rubngc@hotmail.com (R.G.-C.); 2 Le2i FRE2005, CNRS, Arts et Métiers, Univ. Bourgogne Franche-Comté, av. des Plaines de l’Yonne, BP16, 89010 Auxerre CEDEX, France; yvon.voisin@u-bourgogne.fr; 3 Institut Pascal, Université Clermont Auvergne, 63178 Clermont Ferrand, France; abiel@inaoep.mx

**Keywords:** chlorophyll content estimation, image processing, biochemical sensor

## Abstract

This work introduces a new vision-based approach for estimating chlorophyll contents in a plant leaf using reflectance and transmittance as base parameters. Images of the top and underside of the leaf are captured. To estimate the base parameters (reflectance/transmittance), a novel optical arrangement is proposed. The chlorophyll content is then estimated by using linear regression where the inputs are the reflectance and transmittance of the leaf. Performance of the proposed method for chlorophyll content estimation was compared with a spectrophotometer and a Soil Plant Analysis Development (SPAD) meter. Chlorophyll content estimation was realized for *Lactuca sativa *L., *Azadirachta indica*, *Canavalia ensiforme*, and *Lycopersicon esculentum*. Experimental results showed that—in terms of accuracy and processing speed—the proposed algorithm outperformed many of the previous vision-based approach methods that have used SPAD as a reference device. On the other hand, the accuracy reached is 91% for crops such as *Azadirachta indica*, where the chlorophyll value was obtained using the spectrophotometer. Additionally, it was possible to achieve an estimation of the chlorophyll content in the leaf every 200 ms with a low-cost camera and a simple optical arrangement. This non-destructive method increased accuracy in the chlorophyll content estimation by using an optical arrangement that yielded both the reflectance and transmittance information, while the required hardware is cheap.

## 1. Introduction

Due to escalating population growth, food crop demand has increased. To obtain higher-yielding crops, several food producers constantly monitor the crop´s health since it is well-known that stress conditions affect photosynthetic activity, which is closely related to yield. There are variables related to crop health such as chlorophyll fluorescence, chlorophyll, calcium, and nitrogen contents. One variable often used and which has obtained satisfactory results is the chlorophyll content in the leaves. This is due to the high correlation found between chlorophyll content and the health of plants [1]. Several methods for estimating chlorophyll content can be found in the current literature and are based on the transmittance or reflectance of the leaf; nevertheless, chloroplast arrangement in the cells is modified by the intensity, color, and duration of the incident light, which produces variations in the values obtained with measurement devices. Therefore, the analyzed plant must be placed under light-controlled conditions before being measured in order to define the chloroplast arrangement [2] as this yields more accurate results.

Devices such as the SPAD 502 (Soil Plant Analysis Development) of Minolta have been used to estimate chlorophyll contents [3,4,5,6,7]. Its main advantage is that it can provide fast estimations with high accuracy. In general, the SPAD measures the leaf absorbance between 650 and 940 nm [2]. Based on the measured absorbance, a SPAD numerical value proportional to the chlorophyll content is then computed. The main disadvantage of the SPAD system is that it only estimates the absorbance at one point of the leaf under analysis, calculating the chlorophyll content only within a small spatial location on the leaf. To solve this problem, iterative measurements at different spatial locations must be performed. Then, the average value of all the measured points corresponds to the approximated chlorophyll content in the plant leaf being analyzed. 

A vision-based approach is frequently used for chlorophyll content estimation. In this context, vision-based techniques estimate the chlorophyll content using information obtained via image processing algorithms, some of them capturing images using airborne cameras [8,9]. These techniques have the advantage of monitoring large land areas; however, their cost is high, and there is no control for ambient lighting changes that might decrease their accuracy. There are other approaches that capture images at different wavelengths. In particular, multispectral and hyperspectral images taken in a range of 300–1200 nm have been used to analyze the health of food crops [10,11,12,13,14,15,16,17,18,19]. In general, multispectral/hyperspectral-based approaches can obtain relatively high accuracy and real-time processing; unfortunately, they have a high cost and large size. 

Other trends that aim to reduce cost and processing time have been previously proposed by researchers [20,21,22,23,24,25] who have used images with visible spectrum (300–700 nm) information and a single RGB (Red-Green-Blue) color space camera to determine the correlation between chlorophyll and nitrogen content. Several of these approaches have been tested in controlled conditions, and are not well-suited for field conditions. In [26], a robotic system was used to transport the camera along the crop, and five images of the leaves were obtained to reduce noise. The mean of the RGB values was used as a reference to determine lettuce calcium deficiency via statistical computations.

In several cases, multiple images are required making the procedures not suitable for real-time processing. For example, Wiwart et al. [27] required three images of successive nodes for chlorophyll estimation, while Pagola et al. [4] required four images. Finally, high accuracy and relatively low processing time were achieved using neural networks as proposed by Odabas et al. and Vesali et al. [28,29], where the leaf being analyzed was placed in front of a smartphone camera and the color transmittance was used as the base for the chlorophyll estimation.

The objective of this work was to propose an accurate, low-cost, and real-time approach for chlorophyll estimation. In this paper, reflectance and transmittance in three broad bands (R,G,B) were proposed as the base parameters. There are two contributions attributed to this work: the first is a novel mathematical formulation that uses reflectance and transmittance for estimating chlorophyll content that delivers robust and accurate chlorophyll estimations for hydroponic food crops; the second is a novel optical arrangement capable of capturing base parameters (reflectance/transmittance). The proposed approach, which reached high accuracy with low cost, could be a promising solution for the current industrial plantation procedures. Experimental results showed that the proposed algorithm reached over *R*^2^ = 0.97 for a *Lactuca sativa* L. hydroponic crop and achieved a fast estimation of the chlorophyll contents in the plant leaf every 200 ms. 

The rest of this manuscript is organized as follows: Section 2 presents the proposed approach in detail; the experimental results and a comparison to previous works are detailed in Section 3; and finally, Section 4 concludes this manuscript.

## 2. Materials and Methods

To validate the proposed optical configuration, a data set was prepared consisting of different plants from the same crop, each with different health states and different chlorophyll content. A *Lactuca sativa* L. hydroponic crop was studied because of its rapid growth. On this crop, a nutritional solution was applied to obtain different health levels for the plants within the crop. A solution able to provide the daily nutritional macronutrient requirements is typically composed of 4.0810 mg of nitrogen, 0.5531 mg of phosphorus, and 0.4881 mg of potassium. Twenty-seven nutritional solutions were prepared, with the concentration of macronutrients varying among them. For the first one, 100% of the daily requirements of each nutrient were applied: that is, 4.0810 mg of nitrogen, 0.5531 mg of phosphorus, and 0.4884 of potassium. For the second and third solutions, nitrogen was reduced to 2.0405 mg and 0.0 mg—corresponding to 50% and 0% of the daily requirement, respectively—while the concentrations of phosphorus and potassium were maintained at 0.5531 mg and 0.4884 mg, respectively. This procedure was repeated for each component of the solution. Each nutritional solution was applied to three different plants, and the experiment was replicated six times. The hydroponic system was placed in a greenhouse with a controlled temperature. The temperature was set to 26 °C during the day and 18 °C at night. For the first 10 days, only water was applied. From Day 11 to 60, 450 mL of solution was applied every third day. Finally, leaf samples were taken after 50 days, when the plant had reached maturity and deterioration due to age was minimal. Chlorophyll content estimation was performed using a SPAD meter (SPAD 502) that computes the optical density difference at two wavelengths: 650 nm and 940 nm. The measurement area of the device was 2 mm × 3 mm. Eight measurements were taken from different zones of the leaf and the mean of measurements was retained as the chlorophyll content value.

For the second experiment, fresh leaves of *Lactuca sativa *L., *Azadirachta indica *L., *Lycopersicum sculentum *L., and *Canavalia ensiforme *L. were collected. Thirteen leaves grouped in three classes according to their greenness, from yellowish to dark green, were used. In a mortar, 0.5 g of fresh plant material was placed and macerated. The whole process was carried out in a place with low luminosity. From the macerate, 4 mL of 99% acetone was mixed with 2 mL ethanol (2:1 v/v), placed in 10 mL tubes, and mixed for 1 min stirring, ensuring complete contact of the plant material. These were then left to stand for 30 min in the freezer in the dark, and centrifuged for 10 min at 2000 rpm. They were covered with aluminum foil and 5 mL of acetone/ethanol (2:1 v/v) was added and stirred for 1 min. Absorbance readings were performed at wavelengths of 663 nm and 645 nm. The control was acetone/ethanol (2:1 v/v).

The obtained values were substituted in the following formulas, described in [30], for the estimation of photosynthetic pigments.

Chlorophyll a (mg/g) = (12.7 * A663) − (2.59 * A645)
(1)

Chlorophyll b (mg/g) = (22.9 * A645) − (4.7 * A663)
(2)

Chlorophyll total (mg/g) = (8.2 * A663) + (20.2 * A645)
(3)

where A663 and A645 are the absorbance measured from 663 nm and 645 nm, respectively. The spectrophotometer was adjusted to zero using the acetone/ethanol mixture.

### 2.1. Acquisition Technique and Base Parameters

Previous work has demonstrated that there are two crucial issues in the chlorophyll estimation procedures. The first is related to the acquisition technique used to capture base parameters. As reported in [2], several acquisition parameters such as the type of light emitted, intensity, or duration from the source and the leaf side (adaxial or abaxial) being illuminated can affect the reflectance and transmittance data. In general, acquisition techniques sensitive to noise yield poor results in terms of accuracy. In addition, environmental conditions such as rain or dust can affect their performance [31]. The second issue relates to the base parameters used in the chlorophyll estimation process. Robust base parameters should deliver accurate results. Nevertheless, previous vision-based approaches have demonstrated that it is difficult to obtain robust parameters for chlorophyll content estimation [32].

For the acquisition technique, previous vision-based approaches have reached low-cost solutions, while compromising accuracy. This is because accuracy is highly related to the environmental conditions during the image acquisition procedures. Therefore, computer vision systems using natural illumination tend to deliver poor performance in terms of accuracy. In this work, we proposed that one alternative to decrease noise (induced by the natural illumination) could be through the use of artificial light sources with known parameters of the light spectrum, thus improving the performance of the chlorophyll estimation. As a result, a novel portable device suitable for chlorophyll estimations was proposed, which uses controlled illumination conditions that help reduce the noise. Furthermore, chlorophyll estimation is performed within a closed environment, thus eliminating environmental perturbations. This means that the presented device is capable of provide health indicators with low noise sensitivity and without environmental perturbations. Prior to the image acquisition stage, all the samples were placed in darkness to define the chloroplast arrangement and to reduce the error in the chlorophyll content estimation.

For the base parameters, previous works have proposed several indices that relate chlorophyll content to the color components in the leaf being analyzed [5,19]. In this work, two different base parameters dependent on the value of each channel of color reflected or transmitted in the RGB space were proposed. Both parameters were obtained simultaneously by using a relatively simple optical configuration. As a result, it was possible to obtain accurate and economic chlorophyll content estimations.

### 2.2. Image Acquisition

**Figure 1:**
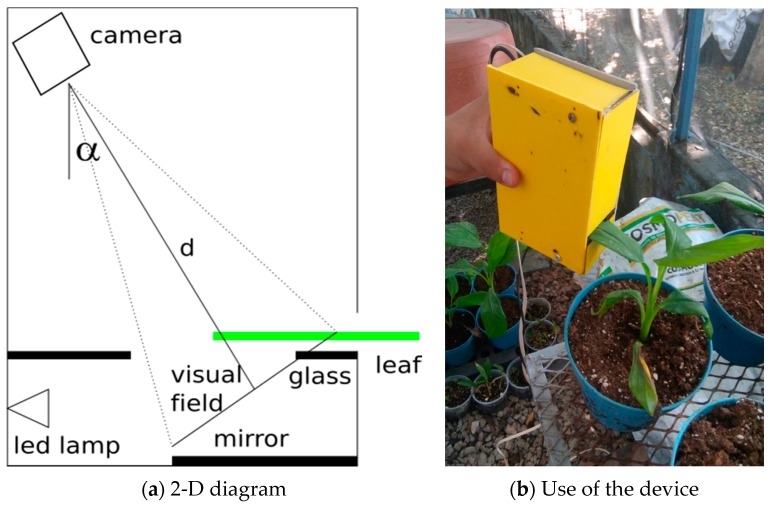
Optical system for the image acquisition: (**a**) Schematic view, (**b**) optical system test.

To capture the base parameters, an optical arrangement was proposed, as seen in Figure 1. A color camera (model DBK 31AU03) was used to provide a 1024 × 768 Bayer pattern image. In a Bayer image, only one of the three color components is obtained. The remaining components are computed by interpolation. A Sony ICX204AK sensor shot the image in the RAW format in the ‘grbg’ Bayer pattern with 8 bits per pixel. 

**Figure 2:**
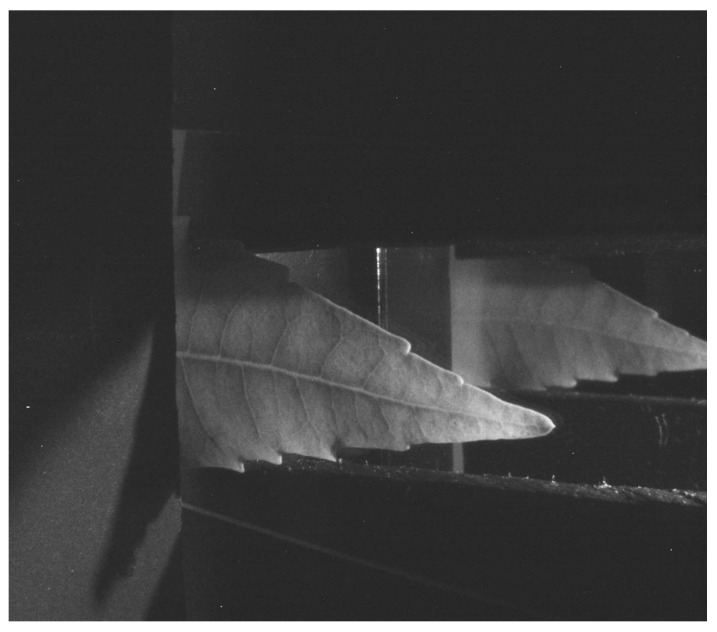
Image of the adaxial and abaxial leaf side in Bayer format.

The camera sensor was a Sony ICX204 with dimensions of 5.8 × 4.92 mm^2^, and the lens used had an 8 mm focal length. The image acquisition device used a MCWHD2 led lamp (800 mW) as the light source. The nominal wavelength spectrum was 400–700 nm, and the maximum irradiance measured at a distance of 200 mm was about 121 µmol m^−2^s^−1^. In addition, the optical arrangement used a 50 × 70 mm^2^ glass that held the leaf to be analyzed. Finally, at the bottom, a 70 mm square mirror reflected the leaf reflectance information. Black-matte plastic pieces were used to control the light flow inside the device and reduce the entry of external light, which would otherwise induce measurement errors. The camera was placed at a 39° horizontal inclination (to observe simultaneously both the upper and lower part of the leaf), with a 100 mm vertical distance and a horizontal gap of 20 mm with respect to the glass in the center. The viewing angle—which determines the longitude of the visual field as a function of the distance between the camera and the glass where the leaf is placed—is calculated by using Equation (4), where *α* is the vertical or horizontal viewing angle, *s* is the vertical or horizontal dimension, and *f* is the focal distance. Using this configuration, both the glass and the mirror could be observed. As a result, the bisector *d* (Figure 1) represents the necessary distance between the vision field and the camera, and is calculated by Equation (5), where *d* is the bisector and *l* is the visual field longitude. The camera was focused on the visual field line, and the diaphragm opening was adjusted to *f*  =  *f*/8. Finally, to acquire the images, a small part (about 2 cm) of the lettuce leaves were inserted in the optical system and the images were acquired in RAW format, as shown in Figure 2.

(4) $$\alpha =2\times \tan^{-1}(s/\left(2\times f\right)$$

(5) $$d=l/\left(2\times \tan\left(\frac{\alpha }{2}\right)\right)$$

As can be observed in Figure 2, the obtained image shows both the adaxial leaf side in the left part of the image and the abaxial leaf side in the right part of the image. The left part of the image produces the transmittance information, while the right part yields the reflectance information. The shape of both leaf sides is not similar due to the perspective distortion. A non-flat leaf must be placed between two glasses to help introduce the leaf in the device. 

### 2.3. Image Processing 

The image processing algorithm is outlined as follows.

1. Convert the Bayer image to an RGB image *I(x*,*y*,3).
2. Calibrate the system using the Macbeth Color Checker table.
3. Compute the binary image (*Ib*(*x*,*y*)) from the green color using active contours.
4. Compute the reflectance information by using the right part of the *Ib’*(*x*,*y*) image, and the transmittance information by using the left part of the *Ib*’(*x*,*y*).

**Figure 3:**
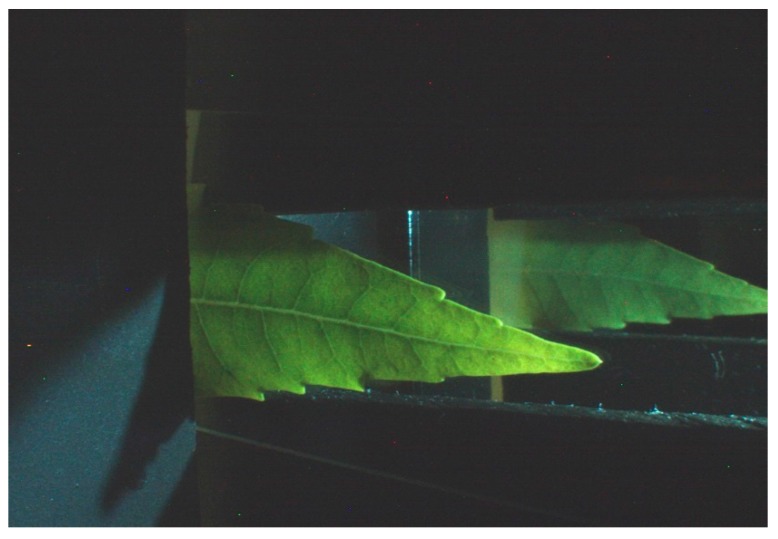
Image in RGB format.

Each one of the steps was developed as follows: the camera produced a Bayer image where only one color channel was provided for each pixel, and the other two color channels were obtained by interpolation. In this case, the input Bayer image (Figure 2) was processed by the Homogeneous edge direct algorithm [33] to obtain an RGB image, as shown in Figure 3. The color calibration of the camera was performed by using a linear model and the Macbeth Color Checker table as described in [34]. 

**Figure 4:**
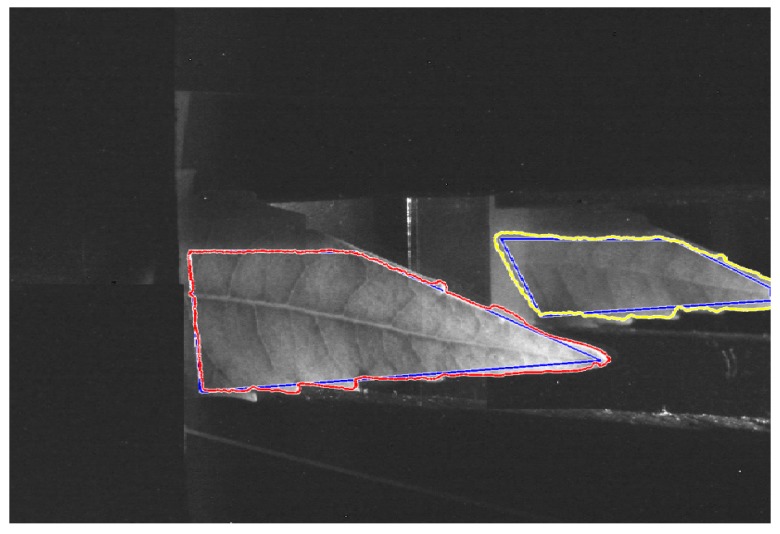
Leaf and background, separation process.

To separate the leaf from the background a method based on active contours has been used [35]. In Figure 4, the results of the binarization are shown. The blue line specifies the initial state of the active contour and the boundaries of the object are indicated in red and yellow color. 

The right part of Figure 3 contains the reflectance information (*Ibr*(*x*, *y*)) and the left part contains the transmittance (*Ibt*(*x*, *y*)). The proposed method computed the reflectance (*Rc*) and transmittance (*Tc*) as shown in Equations (6) and (7), where *c*  =  [*R*, *G*, *B*], *I*(*x*, *y*, *c*) is the input RGB image (Figure 3), and *Ibr*(*x*, *y*), *Ibt*(*x*, *y*) are the binary images corresponding to the reflectance and transmittance, respectively; as seen in Figure 4b.

(6) $$Rc=(\overset{c}{\underset{1}{\sum }}(I\left(x,y,c\right)\times \;Ibr\left(x,y\right)))/n$$

(7) $$Tc=(\overset{c}{\underset{1}{\sum }}(I\left(x,y,c\right)\times \;Ibt\left(x,y\right)))/n\;$$

### 2.4. Chlorophyll Content Estimation by Linear Regression

An association between the base parameters (*Rc* and *Tc*) and chlorophyll content was obtained by linear regression, providing high performance in terms of accuracy and processing time. In general, the main contribution of this work was the mathematical formulation, which used reflectance/transmittance as the base parameters, as well as basic image processing algorithms and a simple linear regression method. As a result, this formulation should provide accurate measurements with low cost and a compact system design.

## 3. Results

**Figure 5:**
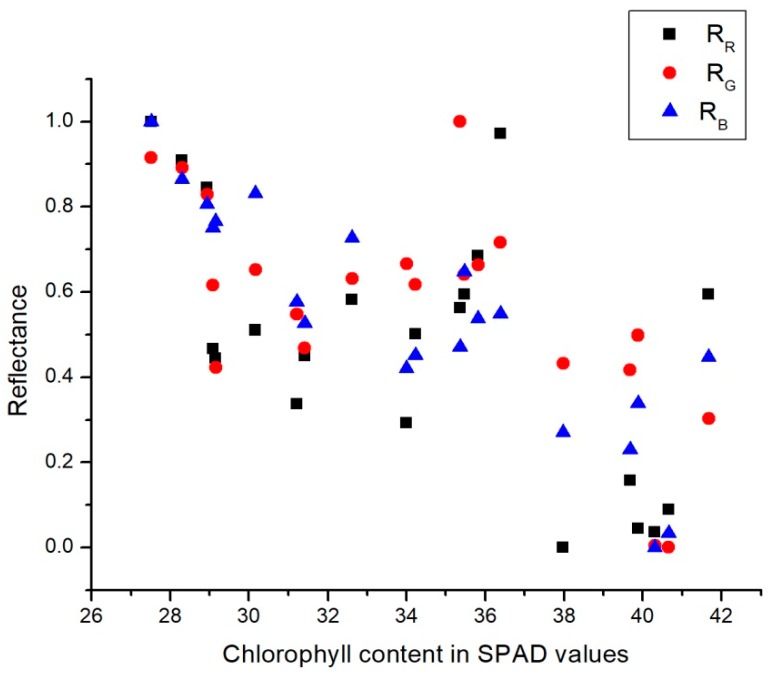
Reflectance according to the chlorophyll content in Soil Plant Analysis Development (SPAD) values.

**Figure 6:**
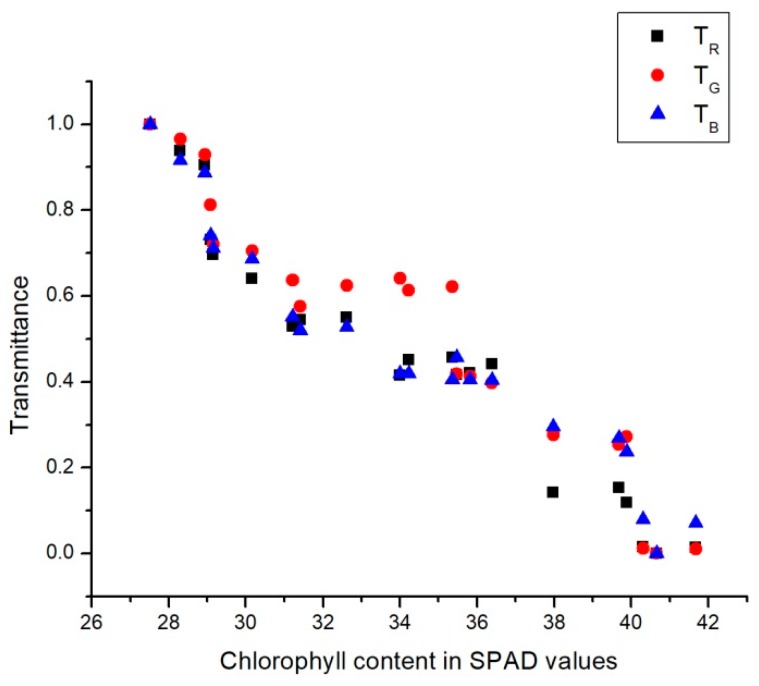
Transmittance according to the chlorophyll content in SPAD values.

MATLAB 2015b was used to perform all image processing steps and the linear regression. Figure 5 and Figure 6 present the chlorophyll contents in SPAD values in relation with the average values for transmittance and reflectance values for each of the R, G, and B channels.

On one hand, as can be seen in Figure 5, reflectance decreased in all channels as the amount of chlorophyll increased. This was because higher chlorophyll contents tends to absorb more energy in the form of light. On the other hand, Figure 6 shows that higher chlorophyll contents reduced the transmittance value for all the channels. This was due to the higher chlorophyll contents which reduced the light that passed through the leaf. 

To build a chlorophyll estimation framework, any statistical regression model has to fulfill the linear dependence of chlorophyll with respect to the reflectance/transmittance indicators. In this work, linear regression was used because of its mathematical simplicity, which involves high processing speed with low computational requirements. It was tested with different combinations of the base parameters proposed in this work. It was demonstrated that all the proposed parameters delivered high accuracy in terms of chlorophyll estimation; in particular, the combination of all of them provided accuracy superior to most of the previous vision-based approaches, and were similar to the SPAD-based measurements. Table 1 shows the results of using the simple linear regression models for each value showed in Figure 5 and Figure 6. The resulting expression for estimating the SPAD values is

SPAD = 40.87 − 14.59 × *T_r_* (8)

The maximum *R*^2^  =  0.94 was for the red channel transmittance *Tr*, and the smallest standard deviation obtained *SD*  =  1.19 SPAD was also for *T_r_*. Even if the normalized root mean square error (NRMSE) value for *T_r_* was 0.2817, the best variable for estimating the chlorophyll content was the transmittance in the red channel.

In Table 2, the results of applying the multiple linear regression models (using two variables) for each value presented in Figure 5 and Figure 6 are shown. The resulting expression for estimating the SPAD values is

SPAD = 40.34 + 4.14 × *R_r_* − 17.65 × *T_r_* (9)

The best *R*^2^  =  0.97 was for the combination of reflectance and transmittance in the red channel (*R_r_*, *T_r_)*. In addition, this combination had the best standard deviation *SD*  =  0.83 SPAD. However, the best NRMSE  =  0.25 was for reflectance and transmittance in the green channel (*R_b_*, *T_b_)*, which had an *R*^2^  =  0.90, and a SD close to the unit. Since multiple linear regression requires more computational power than simple linear regression, it was possible to conclude that a single variable model was one of the best options for chlorophyll estimations in terms of simplicity. Simple linear regression using transmittance as a health indicator combined both relatively high accuracy (*R*^2^ = 0.93) and a very simple mathematical formulation.

Thirteen plants were also used to evaluate the proposal where the chlorophyll content was determined using a spectrophotometer, as shown in Table 3. The mean and the standard deviation of the chlorophyll content, the *R*^2^, and the NRMSE of the chlorophyll estimation using linear regression are shown.

Relationships between the chlorophyll content and transmittance values were determined by linear regression yielding Equations (10)–(18). *T_r_* has been used because it has been demonstrated previously that it yields the most reliable performance to chlorophyll content estimation.

Chlorophyll a (*Canavalia ensiforme* leaves) = 51.64 − 161.71 × *T_r_* (10)

Chlorophyll b (*Canavalia ensiforme* leaves) = 19.63 − 49.51 × *T_r_* (11)

Total chlorophyll (*Canavalia ensiforme* leaves) = 69.97 − 203.01 × *T_r_* (12)

Chlorophyll a (*Azadirachta indica* leaves) = 27.21 − 49.03 × *T_r_* (13)

Chlorophyll b (*Azadirachta indica* leaves) = 10.76 − 20.31 × *T_r_* (14)

Total chlorophyll (*Azadirachta indica* leaves) = 37.97 − 69.35 × *T_r_* (15)

Chlorophyll a (*Lycopersicon esculentum* leaves) = 114.41 − 862.14 × *T_r_* (16)

Chlorophyll b (*Lycopersicon esculentum* leaves) = 37.95 − 281.80 × *T_r_* (17)

Total chlorophyll (*Lycopersicon esculentum* leaves) = 152.31 − 1143.50 × *T_r_* (18)

Like other methods based on computer vision, the equation used for the estimation of chlorophyll for each crop is different. On the other hand, the values of *R*^2^ obtained for *Azadirachta indica* and *Lycopersicon esculentum* leaves showed that this proposal was adequate for chlorophyll content estimation (a, b, and total). Nevertheless, the lowest values of *R*^2^ obtained for the *Canavalia ensiforme* leaves showed that non-flat leaves introduced errors into the chlorophyll content estimation. The performance of the chlorophyll content estimation can be improved by using a more robust method to select pixels used in the estimation process, or by implementing a method to increase flatness of the leaf.

### 3.1. Processing Speed and System Size

In Table 4, the processing speeds for the different versions of the proposed algorithm are presented. Clearly, the increase in the number of variables used in the regression model also increased the processing time. Nevertheless, the proposed algorithm reached real-time processing. Regarding the system size, the device used was as illustrated in Figure 1, which was a portable and small device (100 mm × 200 mm). Currently, the device must be connected to a computer that hosts the image processing algorithms and the chlorophyll content estimation algorithm. Due to the simplicity of the proposed approach, however, it can be implemented in either a small processor embedded in the optical device, or in a smart camera. The developed device can then maintain the same size, and be a promising solution for portable chlorophyll content estimation.

### 3.2. Comparison with Previous Work

Let *F* (*R_r_*, *T_r_*) be the best performance in terms of accuracy. In Equation (9), the corresponding adjusted model is presented, where *F* is the estimated chlorophyll value while *R_r_* and *T_r_* are the base parameters in the mathematical formulation. In Table 5, the accuracy comparisons with respect to previous vision-based approaches are shown. This method has been compared to previous works that have used SPAD measurements as reference values. In the case of multiple crops in the same approach, we considered the best *R*^2^ value. As observed, the proposed measurement approach outperformed most of the previous vision-based approaches because previous formulations used base parameters that had low stability under illumination changes. For this work, given the wide availability of low-cost RGB cameras, the proposed solution could be a promising chlorophyll estimation framework with a similar accuracy and size as SPAD, but with a lower cost and lower processing time (near 200 ms). As a work in progress, we will apply the proposed approach to other hydroponic crops (*Raphanus sativus*, *Coriandrum sativum*, and *Brassica oleracea*).

## 4. Conclusions

In this work, a novel chlorophyll estimation framework was proposed based on an optical arrangement capable of capturing base parameters (reflectance/transmittance). It was demonstrated that reflectance/transmittance delivered robust and discriminant values for the chlorophyll content estimations. The proposed optical arrangement captured the reflectance and transmittance changes in the leaf, analyzing them simultaneously and with low cost. The experimental results were encouraging: they demonstrated that a multiple linear regression algorithm using two variables achieved 97% accuracy for the hydroponic crop analyzed using a chlorophyll estimation method such as SPAD, while reaching estimated values close to the real values obtained with the spectrophotometer. In addition, the proposed approach delivered fast measurements with low cost and allowed a compact system design. To determine if other crop types present similar patterns, the mathematical model can be applied to several different food crops. In any case, the acquisition device and structure of the learning algorithm have to remain the same, therefore, only a color comparison of the crop being analyzed and an adjustment of the multiple regression models are needed.

For the developed device, the initial formulation was made by using the full area of the sample. However, the experimental results demonstrated that a similar performance could be obtained with small regions of the leaf being analyzed. Thus, the measurement device size could be smaller than in the presented version. As a work in progress, a smart camera is currently being developed. The chlorophyll values could then be displayed on a compact device, which would be useful for farmers and the current industrial plantation procedures.

## Figures and Tables

**Table 1:** Regression models (simple linear regression).

Independent Variables	*R*^2^	Standard Deviation (SPAD)	NRMSE
*R_r_*	0.78	1.69	0.05
*R_g_*	0.80	2.60	0.07
*R_b_*	0.76	2.27	0.05
*T_r_*	0.94	1.19	0.28
*T_g_*	0.91	1.35	0.19
*T_b_*	0.92	1.30	0.26

**Table 2:** Multiple linear regression models (using two variables).

Independent Variables	*R*^2^	Standard Deviation (SPAD)	NRMSE
*R_r_*, *T_r_*	0.97	0.83	0.36
*R_g_*, *Tg*	0.96	0.94	0.43
*R_b_*, *T_b_*	0.92	1.34	0.25

**Table 3:** Chlorophyll a, b and total content in *Canavalia ensiforme*, *Azadirachta indica*, and *Lycopersicon esculentum* leaves.

	*Canavalia ensiforme* Leaves			*Azadirachta indica* Leaves			*Lycopersicon esculentum* Leaves		
	Mean, std	*R*^2^	NRMSE	Mean, std	*R*^2^	NRMSE	Mean, std	*R*^2^	NRMSE
*T_r_* (%)	0.16 ± 0.07			0.21 ± 0.09			0.29 ± 0.05		
Chlorophyll a (µg/mL)	26.07 ± 14.02	0.73	0.01	16.47 ± 4.34	0.91	0.02	21.97 ± 3.37	0.96	0.02
Chlorophyll b (µg/mL)	11.80 ± 4.63	0.63	0.03	6.31 ± 1.73	0.98	0.30	8.12 ± 0.84	0.99	0.03
Chlorophyll b}total	37.86 ± 18.49	0.66	0.15	22.77 ± 6.06	0.94	0.02	30.08 ± 3.53	0.97	0.03

**Table 4:** Processing speed of the proposed algorithm.

Case	Processing Time (ms)
*F* (*T_r_*)	150
*F* (*R_g_*, *Tr*)	188

**Table 5:** Vision-based approaches for chlorophyll content estimation.

Approach	Accuracy (*R*^2^)
H. Noh and Q. Zhang (2012), Whole area	0.86
H. Noh and Q. Zhang (2012), Bright area	0.87
H. Noh and Q. Zhang (2012), Corn area	0.85
Tewari et al. (2013)	0.94
Hao Hu et al. (2014), Green Value	0.74
Hao Hu et al. (2014), Red Value	0.75
Pagola et al. (2009), IpcaM4	0.92
Pagola et al. (2009), IpcaM2	0.92
Moghaddam et al. (2011), MLPN	0.94
Moghaddam et al. (2011), R, B (regression)	0.88
Kawashima et al. (1998), NORMALIZED ‘r’	0.79
Kawashima et al. (1998), NORMALIZED ‘g’	0.76
This work, *F* (*R_r_*, *T_r_*)	0.97
